# Supplementary material for: Plasma Phospholipid Fatty Acids and Risk of Venous Thromboembolism: Mendelian Randomization Investigation
Source: Nutrients. 2022 Aug 16;14(16):3354. doi: 10.3390/nu14163354 (PMC9412533; doi:10.3390/nu14163354)
Supplement: Supplementary file 1 [file nutrients-14-03354-s001.zip › nutrients-1838079-supplementary.pdf]

Supporting information for

Plasma phospholipid fatty acids and risk of venous thromboembolism: Mendelian randomization investigation

Shuai Yuan, Xue Li, Pierre—Emmanuel Morange, Maria Bruzelius, Susanna C. Larsson

Supplementary Table 1. Genetic instrumental variables for ten plasma phospholipid fatty acids
Supplementary Table 2. Supplementary genetic instrumental variables for arachidonic acid
Supplementary Table 3. Data used in the analyses for venous thromboembolism in the International Network Against Venous Thrombosis (INVENT) Consortium
Supplementary Table 4. Data used in the analyses for venous thromboembolism and two subtypes in the FinnGen study
Supplementary Table 5. Power estimation
Supplementary Table 6. F statistics of used genetic instruments
Supplementary Table 7. Associations after Benjamini—Hochberg correction (the false discovery rate, FDR)
Supplementary Table 8. Sensitivity analyses for genetically predicted palmitoleic acid and arachidonic acid

Table S1. Genetic instrumental variables for ten plasma phospholipid fatty acids.

Type of FA	FA	SNP	Chr	Nearby gene	EA	% variance explained	Beta[a]	SE	*P* value
n—3 PUFA	ALA	rs174547	11	*FADS1*	C	1.0	0.02	0.001	3.50×10^{-64}
n—3 PUFA	EPA	rs3798713	6	*ELOVL2*	C	0.4	0.04	0.005	1.90×10^{-12}
n—3 PUFA	EPA	rs174538	11	*FADS1*	G	1.7	0.08	0.005	5.40×10^{-58}
n—3 PUFA	DPA	rs780094[b]	2	*GCKR*	T	0.5	0.02	0.003	9.00×10^{-9}
n—3 PUFA	DPA	rs3734398	6	*ELOVL2*	C	2.7	0.04	0.003	9.70×10^{-43}
n—3 PUFA	DPA	rs174547	11	*FADS1*	T	8.4	0.08	0.003	3.80×10^{-154}
n—3 PUFA	DHA	rs2236212	6	*ELOVL2*	G	0.7	0.11	0.014	1.30×10^{-15}
n—6 PUFA	LA	rs10740118	10	*JMJD1C*	G	0.2—0,7	0.25	0.050	8.10×10^{-9}
n—6 PUFA	LA	rs174547	11	*FADS1*	C	7.6—18.1	1.47	0.050	5.00×10^{-274}
n—6 PUFA	LA	rs16966952	16	*NTAN1*	G	0.5—2.5	0.35	0.040	1.20×10^{-15}
n—6 PUFA	AA	rs174547	11	*FADS1*	T	3.7—37.6	1.69	0.020	3.3×10^{-971}
n—6 PUFA	AA	rs16966952	16	*NTAN1*	G	0.1—0.6	0.20	0.030	2.40×10^{-10}
n—7 MUFA	POA	rs780093[b]	2	*GCKR*	T	0.2—0.9	0.02	0.003	9.80×10^{-10}
n—7—MUFA	POA	rs6722456	2	*RN7SKP93*	G	0.01—0.6	0.05	0.009	4.10×10^{-8}
n—7 MUFA	POA	rs603424	10	*SCD*	G	0.3—1.6	0.03	0.004	5.70×10^{-15}
n—7 MUFA	POA	rs11190604	10	*HIF1AN*	G	0.02—0.7	0.02	0.004	5.70×10^{-9}
n—7 MUFA	POA	rs102275	11	*FADS1/2*	C	0.15—1.0	0.02	0.003	6.60×10^{-13}
n—9 MUFA	OA	rs102275	11	*FADS1/2*	C	0.3—2.1	0.23	0.020	2.20×10^{-32}
SFA	PA	rs2391388	1	*ALG14*	C	0.2—1.0	0.18	0.030	2.70×10^{-11}
SFA	SA	rs6675668	1	*ALG14*	G	0.4—1.4	0.17	0.020	2.20×10^{-18}
SFA	SA	rs11119805	1	*LPGAT1*	T	0.01—0.7	0.17	0.030	2.80×10^{-9}
SFA	SA	rs102275	11	*FADS1/2*	T	0.3—1.2	0.18	0.020	1.30×10^{-20}

AA indicates arachidonic acid; ALA, α—linolenic acid; Chr, chromosome; DHA, docosahexaenoic acid; DPA, docosapentaenoic acid; EA, effect allele; EPA, eicosapentaenoic acid; FA, fatty acid; LA, linoleic acid; MUFA, monounsaturated fatty acid; OA, oleic acid; PA, palmitic acid; POA, palmitoleic acid; PUFA, polyunsaturated fatty acid; SA, stearic acid; SE, standard error; SFA, saturated fatty acid; SNP, single—nucleotide polymorphisms.

[a] The beta coefficients represent the change in percentage of total fatty acids for each additional effect allele.

[b] SNPs in *GCKR* gene show multiple pleiotropic associations with potential confounders and were excluded in the sensitivity analysis.

Table S2. Supplementary genetic instrumental variables for arachidonic acid.

SNP	Chr	Nearby gene	EA	EAF	% variance explained	Beta[a]	SE	*P* value
rs12285167	11	*DAGLA*	C	0.20	0.70%	0.284	0.040	1.10×10^{-12}
rs174547	11	*FADS1*	T	0.68	32.40%	1.691	0.025	3.3×10^{-971}
rs17663676	11	*AHNAK*	C	0.97	0.40%	0.469	0.086	4.50×10^{-68}
rs259874	11	*FTH1*	G	0.10	0.80%	0.412	0.072	9.80×10^{-9}
rs2903922	11	*INCENP*	T	0.27	0.60%	0.233	0.034	5.70×10^{-12}
rs3741259	11	*SYT7*	C	0.10	0.50%	0.315	0.058	4.50×10^{-8}
rs472031	11	*FADS3*	A	0.10	1.20%	0.510	0.047	2.30×10^{-27}
rs760306	11	*BEST1*	C	0.24	0.70%	0.281	0.036	2.40×10^{-15}
rs1741	16	*PDXDC1*	G	0.31	0.40%	0.201	0.031	1.60×10^{-10}

Chr, chromosome; EA, effect allele; EAF, effect allele frequency; SE, standard error; SNP, single-nucleotide polymorphisms.
[a] The beta coefficients represent the change in percentage of total fatty acids for each additional effect allele.

Table S3. Data used in the analyses for venous thromboembolism in the International Network Against Venous Thrombosis (INVENT) Consortium.

								Levels of fatty acid			Venous thromboembolism		
**Exposure**	**SNP**	**Chr**	**Position**	***Gene***	**EA**	**NEA**	**EAF**	**Beta**	**SE**	***P* value**	**Beta**	**SE**	***P* value**
ALA	rs174547	11	61570783	*FADS1*	C	T	0.33	0.020	0.001	3.50×10^{-64}	-0.045	0.010	1.58×10^{-5}
EPA	rs174538	11	61560081	*C11orf10*	G	A	0.70	0.080	0.005	5.40×10^{-58}	0.042	0.011	9.03×10^{-5}
EPA	rs3798713	6	11008622	*ELOVL2*	C	G	0.42	0.035	0.005	1.90×10^{-12}	-0.008	0.010	0.426
DPA	rs3734398	6	10982973	*ELOVL2*	C	T	0.42	0.040	0.003	9.70×10^{-43}	-0.009	0.010	0.387
DPA	rs174547	11	61570783	*FADS1*	T	C	0.67	0.075	0.003	3.80×10^{-154}	0.045	0.010	1.58×10^{-5}
DHA	rs2236212	6	10995015	*ELOVL2*	G	C	0.59	0.110	0.014	1.30×10^{-15}	0.008	0.010	0.428
LA	rs10740118	10	65101207	*JMJD1C*	G	C	0.58	0.250	0.050	8.10×10^{-9}	-0.012	0.010	0.218
LA	rs16966952	16	15135943	*PDXDC1*	G	A	0.71	0.350	0.040	1.20×10^{-15}	0.023	0.011	0.033
LA	rs174547	11	61570783	*FADS1*	C	T	0.33	1.470	0.050	5.00×10^{-274}	-0.045	0.010	1.58×10^{-5}
AA	rs16966952	16	15135943	*PDXDC1*	G	A	0.71	0.200	0.030	2.40×10^{-10}	0.023	0.011	0.033
AA	rs174547	11	61570783	*FADS1*	T	C	0.67	1.690	0.020	3.30×10^{-971}	0.045	0.010	1.58×10^{-5}
AA_supp	rs12285167	11	61492039	*DAGLA*	C	A	0.79	0.284	0.040	1.05×10^{-12}	0.022	0.012	0.073
AA_supp	rs1741	16	15130351	*PDXDC1*	G	C	0.71	0.201	0.031	1.64×10^{-10}	0.026	0.011	0.017
AA_supp	rs17663676	11	62201002	*AHNAK*	C	T	0.04	0.469	0.086	4.52×10^{-8}	0.026	0.026	0.314
AA_supp	rs259874	11	61811441	*FTH1*	G	A	0.86	0.412	0.072	9.79×10^{-9}	0.002	0.016	0.922
AA_supp	rs2903922	11	61929298	*INCENP*	T	A	0.71	0.233	0.034	5.70×10^{-12}	0.004	0.011	0.709
AA_supp	rs3741259	11	61282350	*SYT7*	C	T	0.90	0.315	0.058	4.50×10^{-8}	0.014	0.017	0.421
AA_supp	rs472031	11	61638420	*FADS3*	A	G	0.09	0.510	0.047	2.34×10^{-27}	0.024	0.017	0.167
AA_supp	rs760306	11	61724292	*BEST1*	C	T	0.73	0.281	0.036	2.42×10^{-15}	0.028	0.011	0.013
AA_supp	rs174547	11	61570783	*FADS1*	T	C	0.67	1.691	0.025	3.30×10^{-971}	0.045	0.010	1.58×10^{-5}
POA	rs11190604	10	102302457	*HIF1AN*	G	A	0.21	0.020	0.004	5.70×10^{-9}	-0.002	0.012	0.845
POA	rs603424	10	102075479	*PKD2L1*	G	A	0.79	0.030	0.004	5.70×10^{-15}	-0.003	0.013	0.804
POA	rs6722456	2	134529091	*RN7SKP93*	G	A	0.97	0.050	0.009	4.10×10^{-8}	0.012	0.033	0.712
POA	rs102275	11	61557803	*FADS1/2*	C	T	0.35	0.020	0.003	6.60×10^{-13}	-0.045	0.010	1.31×10^{-5}
OA	rs102275	11	61557803	*FADS1/2*	C	T	0.35	0.230	0.020	2.20×10^{-32}	-0.045	0.010	1.31×10^{-5}
PA	rs2391388	1	95485825	*ALG14*	C	A	0.46	0.180	0.030	2.70×10^{-11}	0.007	0.010	0.464
SA	rs6675668	1	211918244	*LPGAT1*	T	A	0.87	0.170	0.030	2.80×10^{-9}	0.038	0.015	0.010
SA	rs11119805	1	95515637	*ALG14*	G	T	0.50	0.170	0.020	2.20×10^{-18}	-0.001	0.010	0.894
SA	rs102275	11	61557803	*FADS1/2*	T	C	0.65	0.180	0.020	1.30×10^{-20}	0.045	0.010	1.31×10^{-5}

AA indicates arachidonic acid; AA_supp, supplementary instruments for arachidonic acid; ALA, α-linolenic acid; DHA, docosahexaenoic acid; DPA, docosapentaenoic acid; DVT, deep vein thrombosis; EA, effect allele; EAF, effect allele frequency; EPA, eicosapentaenoic acid; FA, fatty acid; LA, linoleic acid; OA, oleic acid; NEA, non-effect allele; PA, palmitic acid; POA, palmitoleic acid; SA, stearic acid; SNP, single nucleotide polymorphism.

Table S4. Data used in the analyses for venous thromboembolism and two subtypes in the FinnGen study.

						FA levels			VTE			PE			DVT		
**Exposure**	**SNP**	**Gene**	**EA**	**NEA**	**EAF**	**Beta**	**SE**	***P* value**	**Beta**	**SE**	***P* value**	**Beta**	**SE**	***P* value**	**Beta**	**SE**	***P* value**
ALA	rs174547	*FADS1*	C	T	0.41	0.020	0.001	3.50×10^{-64}	-0.065	0.015	1.02×10^{-6}	-0.052	0.021	0.013	-0.082	0.020	4.96×10^{-6}
EPA	rs174538	*C11orf10*	G	A	0.65	0.080	0.005	5.40×10^{-58}	0.052	0.015	0.001	0.048	0.022	0.026	0.057	0.021	0.007
EPA	rs3798713	*ELOVL2*	C	G	0.44	0.035	0.005	1.90×10^{-12}	0.039	0.015	0.008	0.044	0.021	0.037	0.008	0.020	0.678
DPA	rs174547	*FADS1*	T	C	0.59	0.075	0.003	3.80×10^{-154}	0.065	0.015	1.02×10^{-6}	0.052	0.021	0.013	0.082	0.020	4.96×10^{-6}
DPA	rs3734398	*ELOVL2*	C	T	0.46	0.040	0.003	9.70×10^{-43}	0.036	0.015	0.013	0.041	0.021	0.048	0.017	0.020	0.386
DHA	rs2236212	*ELOVL2*	G	C	0.57	0.110	0.014	1.30×10^{-15}	-0.034	0.015	0.019	-0.035	0.021	0.100	-0.006	0.020	0.779
LA	rs10740118	*JMJD1C*	G	C	0.62	0.250	0.050	8.10×10^{-9}	-0.005	0.015	0.722	0.011	0.021	0.596	0.009	0.021	0.657
LA	rs16966952	*PDXDC1*	G	A	0.68	0.350	0.040	1.20×10^{-15}	0.042	0.016	0.007	0.028	0.022	0.204	0.062	0.022	0.004
LA	rs174547	*FADS1*	C	T	0.41	1.470	0.050	5.00×10^{-274}	-0.065	0.015	1.02×10^{-6}	-0.052	0.021	0.013	-0.082	0.020	4.96×10^{-6}
AA	rs16966952	*PDXDC1*	G	A	0.68	0.200	0.030	2.40×10^{-10}	0.042	0.016	0.007	0.028	0.022	0.204	0.062	0.022	0.004
AA	rs174547	*FADS1*	T	C	0.59	1.690	0.020	3.30×10^{-971}	0.065	0.015	1.02×10^{-6}	0.052	0.021	0.013	0.082	0.020	4.96E-05
AA_supp	rs12285167	*DAGLA*	C	A	0.78	0.284	0.040	1.05×10^{-12}	0.025	0.017	0.149	0.002	0.025	0.928	0.047	0.024	0.052
AA_supp	rs1741	*PDXDC1*	G	C	0.69	0.201	0.031	1.64×10^{-10}	0.043	0.016	0.005	0.030	0.022	0.173	0.063	0.022	0.003
AA_supp	rs174547	*FADS1*	T	C	0.59	1.691	0.025	3.30×10^{-971}	0.065	0.015	1.02×10^{-6}	0.052	0.021	0.013	0.082	0.020	4.96×10^{-6}
AA_supp	rs17663676	*AHNAK*	C	T	0.04	0.469	0.086	4.52×10^{-8}	0.030	0.038	0.430	0.010	0.054	0.859	0.005	0.053	0.922
AA_supp	rs259874	*FTH1*	G	A	0.90	0.412	0.072	9.79×10^{-9}	0.031	0.025	0.223	0.003	0.036	0.929	0.064	0.035	0.065
AA_supp	rs2903922	*INCENP*	T	A	0.77	0.233	0.034	5.70×10^{-12}	0.028	0.017	0.104	0.043	0.025	0.079	-0.005	0.024	0.834
AA_supp	rs3741259	*SYT7*	C	T	0.87	0.315	0.058	4.50×10^{-8}	0.047	0.022	0.031	0.049	0.031	0.115	0.052	0.030	0.087
AA_supp	rs472031	*FADS3*	A	G	0.06	0.510	0.047	2.34×10^{-27}	0.045	0.030	0.132	0.065	0.043	0.133	0.038	0.042	0.362
AA_supp	rs760306	*BEST1*	C	T	0.61	0.281	0.036	2.42×10^{-15}	0.002	0.015	0.885	-0.008	0.021	0.713	0.000	0.021	0.996
POA	rs102275	*FADS1/2*	C	T	0.42	0.020	0.003	6.60×10^{-13}	-0.063	0.015	1.75×10^{-6}	-0.047	0.021	0.026	-0.084	0.020	3.69×10^{-6}
POA	rs11190604	*HIF1AN*	G	A	0.21	0.020	0.004	5.70×10^{-9}	-0.035	0.018	0.045	-0.023	0.025	0.366	-0.058	0.024	0.018
POA	rs603424	*PKD2L1*	G	A	0.88	0.030	0.004	5.70×10^{-15}	-0.018	0.023	0.422	-0.019	0.032	0.550	-0.014	0.032	0.655
POA	rs6722456	*RN7SKP93*	G	A	0.99	0.050	0.009	4.10×10^{-8}	0.098	0.098	0.318	0.245	0.139	0.079	-0.006	0.137	0.965
OA	rs102275	*FADS1/2*	C	T	0.42	0.230	0.020	2.20×10^{-32}	-0.063	0.015	1.75×10^{-6}	-0.047	0.021	0.026	-0.084	0.020	3.69×10^{-6}
PA	rs2391388	*ALG14*	C	A	0.37	0.180	0.030	2.70×10^{-11}	0.000	0.015	0.977	0.011	0.021	0.621	-0.010	0.021	0.622
SA	rs102275	*FADS1/2*	T	C	0.58	0.180	0.020	1.30×10^{-20}	0.063	0.015	1.75×10^{-6}	0.047	0.021	0.026	0.084	0.020	3.69×10^{-6}
SA	rs11119805	*LPGAT1*	T	A	0.90	0.170	0.030	2.80×10^{-9}	-0.018	0.024	0.444	-0.040	0.035	0.243	-0.007	0.033	0.828
SA	rs6675668	*ALG14*	G	T	0.61	0.170	0.020	2.20×10^{-18}	0.006	0.015	0.685	-0.006	0.021	0.787	0.016	0.021	0.432

AA indicates arachidonic acid; AA_supp, supplementary instruments for arachidonic acid; ALA, α-linolenic acid; DHA, docosahexaenoic acid; DPA, docosapentaenoic acid; DVT, deep vein thrombosis; EA, effect allele; EAF, effect allele frequency; EPA, eicosapentaenoic acid; FA, fatty acid; LA, linoleic acid; OA, oleic acid; NEA, non-effect allele; PA, palmitic acid; PE, pulmonary embolism; POA, palmitoleic acid; SA, stearic acid; SNP, single nucleotide polymorphism; VTE, venous thromboembolism.

Table S5. Power estimation.

Fatty acid	INVENT	FinnGen (VTE)	FinnGen (PE)	FinnGen (DVT)
α—Linolenic acid (ALA, 18:3*n*—3)	≤0.83 or ≥1.18	≤0.73 or ≥1.28	≤0.61 or ≥1.39	≤0.62 or ≥1.38
Eicosapentaenoic acid (EPA, 20:5*n*—3)	≤0.88 or ≥1.13	≤0.81 or ≥1.19	≤0.73 or ≥1.27	≤0.74 or ≥1.26
Docosapentaenoic acid (DPA, 22:5*n*—3)	≤0.94 or ≥1.06	≤0.92 or ≥1.08	≤0.88 or ≥1.12	≤0.90 or ≥1.11
Docosahexaenoic acid (DHA, 22:6*n*—3)	≤0.79 or ≥1.22	≤0.68 or ≥1.33	≤0.54 or ≥1.47	≤0.54 or ≥1.46
Linoleic acid (LA, 18:2*n*—6)	≤0.95 or ≥1.05	≤0.93 or ≥1.07	≤0.90 or ≥1.10	≤0.91 or ≥1.10
Arachidonic acid (AA, 20:4*n*—6)	≤0.96 or ≥1.04	≤0.94 or ≥1.06	≤0.91 or ≥1.09	≤0.91 or ≥1.09
Palmitoleic acid (POA, 16:1*n*—7)	≤0.88 or ≥1.12	≤0.82 or ≥1.19	≤0.73 or ≥1.27	≤0.74 or ≥1.26
Oleic acid (OA, 18:1*n*—9)	≤0.84 or ≥1.17	≤0.74 or ≥1.26	≤0.62 or ≥1.38	≤0.63 or ≥1.36
Palmitic acid (PA, 16:0)	≤0.78 or ≥1.24	≤0.78 or ≥1.24	≤0.53 or ≥1.48	≤0.54 or ≥1.46
Stearic acid (SA, 18:0)	≤0.88 or ≥1.12	≤0.81 or ≥1.19	≤0.72 or ≥1.27	≤0.73 or ≥1.26

DVT indicates deep vein thrombosis; INVENT, the International Network Against Venous Thrombosis; PE, pulmonary embolism; VTE, venous thromboembolism.
The power estimate was based on 80% power.

Table S6. *F* statistics of used genetic instruments.

Names	INVENT		FinnGen	
	**Sample size**	***F***	**Sample size**	***F***
α—Linolenic acid (ALA, 18:3*n*—3)	202,356	2,043.98	260,405	2,630.33
Eicosapentaenoic acid (EPA, 20:5*n*—3)	202,356	2,170.28	260,405	2,792.87
Docosapentaenoic acid (DPA, 22:5*n*—3)	202,356	1,2632.84	260,405	16,256.82
Docosahexaenoic acid (DHA, 22:6*n*—3)	202,356	1,426.46	260,405	1,835.67
Linoleic acid (LA, 18:2*n*—6)	202,356	1,1716.78	260,405	15,077.99
Arachidonic acid (AA, 20:4*n*—6)	202,356	2,6895.02	260,405	34,610.39
Palmitoleic acid (POA, 16:1*n*—7)	202,356	1,111.53	260,405	1,430.40
Oleic acid (OA, 18:1*n*—9)	202,356	2,457.74	260,405	3,162.79
Palmitic acid (PA, 16:0)	202,356	1,221.45	260,405	1,571.85
Stearic acid (SA, 18:0)	202,356	1,376.54	260,405	1,771.44

INVENT, the International Network Against Venous Thrombosis.

Table S7. Associations after Benjamini—Hochberg correction (the false discovery rate, FDR).

**Exposure**	**INVENT**		**FinnGen (VTE)**		**Combined**		**FinnGen (PE)**		**FinnGen (DVT)**	
	P	***P_FDR***	***P***	***P_FDR***	***P***	***P_FDR***	***P***	***P_FDR***	***P***	***P_FDR***
α—Linolenic acid (ALA, 18:3*n*—3)	1.60×10^{-5}	5.33×10^{-5}	1.03×10^{-5}	3.43×10^{-5}	1.42×10^{-9}	7.12×10^{-9}	1.27×10^{-2}	3.17×10^{-2}	4.95×10-5	1.65×10^{-4}
Eicosapentaenoic acid (EPA, 20:5*n*—3)	1.42×10^{-3}	2.02×10^{-3}	2.77×10^{-5}	5.54×10^{-5}	4.98×10^{-7}	8.30×10^{-7}	3.90×10^{-3}	1.95×10^{-2}	8.07E-03	1.01×10^{-2}
Docosapentaenoic acid (DPA, 22:5*n*—3)	8.40×10^{-4}	1.40×10^{-3}	4.16×10^{-7}	4.16×10^{-6}	$1.76E\times10^{-8}$	4.39×10^{-8}	1.73×10^{-3}	1.73×10^{-2}	6.74×10-5	1.69×10^{-4}
Docosahexaenoic acid (DHA, 22:6*n*—3)	4.30×10^{-1}	4.77×10^{-1}	1.93×10^{-2}	2.14×10^{-2}	$5.07E\times10^{-1}$	5.63×10^{-1}	9.98×10^{-2}	1.25×10^{-1}	7.79E-01	7.79×10^{-1}
Linoleic acid (LA, 18:2*n*—6)	1.07×10^{-4}	2.67×10^{-4}	1.95×10^{-4}	2.79×10^{-4}	1.03×10^{-7}	2.07×10^{-7}	4.13×10^{-2}	6.89×10^{-2}	1.30E-03	2.16×10^{-3}
Arachidonic acid (AA, 20:4*n*—6)	5.86×10^{-6}	5.86×10^{-5}	2.85×10^{-6}	1.42×10^{-5}	8.09×10^{-11}	8.09×10^{-10}	8.85×10^{-3}	2.95×10^{-2}	1.35×10-5	1.35×10^{-4}
Palmitoleic acid (POA, 16:1*n*—7)	2.10×10^{-2}	2.63×10^{-2}	1.30×10^{-4}	2.16×10^{-4}	7.10×10^{-5}	8.87×10^{-5}	8.16×10^{-2}	1.17×10^{-1}	8.31×10-5	1.66×10^{-4}
Oleic acid (OA, 18:1*n*—9)	1.45×10^{-5}	7.25×10^{-5}	1.74×10^{-5}	4.35×10^{-5}	1.69×10^{-9}	5.63×10^{-9}	2.56×10^{-2}	5.12×10^{-2}	3.70×10-5	1.85×10^{-4}
Palmitic acid (PA, 16:0)	4.61×10^{-1}	4.61×10^{-1}	9.77×10^{-1}	9.77×10^{-1}	5.47×10^{-1}	5.47×10^{-1}	6.22×10^{-1}	6.22×10^{-1}	6.22E-01	6.91×10^{-1}
Stearic acid (SA, 18:0)	1.48×10^{-4}	2.96×10^{-4}	4.31×10^{-3}	5.39×10^{-3}	2.36×10^{-6}	3.37×10^{-6}	3.78×10^{-2}	4.20×10^{-2}	1.41E-03	2.01×10^{-3}

Table S8. Sensitivity analyses for genetically predicted palmitoleic acid and arachidonic acid.

				Cochran's	IVW—random effects			Weighted median			MR—Egger regression				
**Source**	**Outcome**	**Fatty acid**	**SNPs**	**Q**	**OR**	**95% CI**	**P**	**OR**	**95% CI**	**P**	**OR**	**95% CI**	**P**	**Intercept**	**P**
INVENT	VTE	Palmitoleic acid (POA, 16:1*n*—7)	4	13.71	0.90	0.73—1.09	0.280	0.98	0.80—1.20	0.849	1.35	0.69—2.61	0.380	-0.058	0.210
INVENT	VTE	Arachidonic acid (AA, 20:4*n*—6)	9	8.95	1.06	1.04—1.09	4.35×10^{-7}	1.06	1.03—1.08	2.07×10^{-6}	1.04	1.01—1.07	0.021	0.013	0.038
INVENT	VTE	Arachidonic acid (AA, 20:4*n*—6)	8	5.95	1.12	1.05—1.18	3.17×10^{-4}	1.10	1.02—1.19	0.018	0.99	0.81—1.21	0.904	0.020	0.214
FinnGen	VTE	Palmitoleic acid (POA, 16:1*n*—7)	4	9.48	0.74	0.56—0.97	0.031	0.73	0.29—1.85	0.513	2.04	0.99—4.21	0.055	-0.132	0.005
FinnGen	VTE	Arachidonic acid (AA, 20:4*n*—6)	9	207.83	1.04	0.99—1.10	0.125	1.08	1.05—1.11	6.44×10^{-9}	1.03	0.97—1.10	0.357	0.006	0.585
FinnGen	VTE	Arachidonic acid (AA, 20:4*n*—6)	8	6.08	1.19	1.09—1.30	8.70×10^{-5}	1.19	1.06—1.34	0.004	1.02	0.74—1.40	0.906	0.025	0.313
FinnGen	PE	Palmitoleic acid (POA, 16:1*n*—7)	4	6.21	0.82	0.6—1.130	0.226	0.82	0.02—43.6	0.921	2.28	0.76—6.90	0.143	-0.133	0.063
FinnGen	PE	Arachidonic acid (AA, 20:4*n*—6)	9	6.82	1.07	1.03—1.12	0.002	1.06	1.01—1.11	0.012	1.04	0.98—1.11	0.176	0.014	0.257
FinnGen	PE	Arachidonic acid (AA, 20:4*n*—6)	8	5.79	1.14	1.00—1.29	0.043	1.04	0.88—1.23	0.654	1.01	0.64—1.60	0.951	0.019	0.603
FinnGen	DVT	Palmitoleic acid (POA, 16:1*n*—7)	4	7.32	0.65	0.46—0.91	0.012	0.60	0.13—2.76	0.516	2.28	0.83—6.26	0.109	-0.163	0.012
FinnGen	DVT	Arachidonic acid (AA, 20:4*n*—6)	9	11.82	1.11	1.06—1.17	5.24×10^{-5}	1.10	1.05—1.15	3.36×10^{-5}	1.07	1.00—1.15	0.044	0.021	0.124
FinnGen	DVT	Arachidonic acid (AA, 20:4*n*—6)	8	9.42	1.22	1.06—1.41	0.006	1.23	1.05—1.45	0.012	1.03	0.60—1.76	0.920	0.027	0.514

CI, confidence interval; DVT indicates deep vein thrombosis; INVENT, the International Network Against Venous Thrombosis; IVW, inverse variance weighted; OR, odds ratio; PE, pulmonary embolism; SNPs, single nucleotide polymorphisms; VTE, venous thromboembolism.
